# The Carpathian range represents a weak genetic barrier in South-East Europe

**DOI:** 10.1186/1471-2156-15-56

**Published:** 2014-05-15

**Authors:** Montserrat Hervella, Neskuts Izagirre, Santos Alonso, Mihai Ioana, Mihai G Netea, Concepción de-la-Rua

**Affiliations:** 1 Department of Genetics, Physical Anthropology and Animal Physiology, University of the Basque Country, Barrio Sarriena s/n 48940, Leioa, Bizkaia, Spain; 2 Department of Medicine, Radboud University Nijmegen Medical Centre, Nijmegen, The Netherlands; 3 University of Medicine and Pharmacy Craiova, Craiova, Romania; 4 Nijmegen Institute for Infection, Inflammation and Immunity (N4i), Radboud University Nijmegen Medical Centre, Nijmegen, The Netherlands

**Keywords:** Mitochondrial DNA, Carpathian Mountains, Romanian population, Genetic barrier

## Abstract

**Background:**

In the present study we have assessed whether the Carpathian Mountains represent a genetic barrier in East Europe. Therefore, we have analyzed the mtDNA of 128 native individuals of Romania: 62 of them from the North of Romania, and 66 from South Romania.

**Results:**

We have analyzed their mtDNA variability in the context of other European and Near Eastern populations through multivariate analyses. The results show that regarding the mtDNA haplogroup and haplotype distributions the Romanian groups living *outside* the Carpathian range (South Romania) displayed some degree of genetic differentiation compared to those living *within* the Carpahian range (North Romania).

**Conclusion:**

The main differentiation between the mtDNA variability of the groups from North and South Romania can be attributed to the demographic movements from East to West (prehistoric or historic) that differently affected in these regions, suggesting that the Carpathian mountain range represents a weak genetic barrier in South-East Europe.

## Background

Assessing the genetic structure of different populations is important for understanding the population history of a certain geographic area from a genetic point of view. It is also important from the epidemiological point of view to avoid spurious association between genetic markers and certain diseases [1,2]. The genetic structure of European populations has been the focus of several recent studies which have identified that geographical distance is more important than cultural and linguistic affinities when explaining the genetic distance between populations [3,4].

While most of the genetic studies of European populations have focused on the Western part of the continent, few studies have assessed the genetic structure of Eastern Europe. Among these, one study assessing the variability of the Y-chromosome has proposed that, although the population structure of the Carpathian basin is relatively homogenous, the Carpathian range from the present territory of Romania represents a genetic barrier between populations living in the East and West of this barrier and mountain populations encompassed within the Carpathian arch [5]. In this study, those populations outside the Carpathian range seemed to show more genetic similarities with East Europe and the Mediterranean regions, while the populations living within the Carpathian range in Transylvania were closer to Central and Western European populations [5]. Other Y-chromosome studies have proposed a homogenous structure of populations in the Balkans [6] or the Dnieper-Carpathian basin [7], but these studies have only analysed Romanian populations from outside the Carpathian range. To our knowledge, no studies have been performed to assess the mtDNA population structure in Romanians.

**Figure 1:**
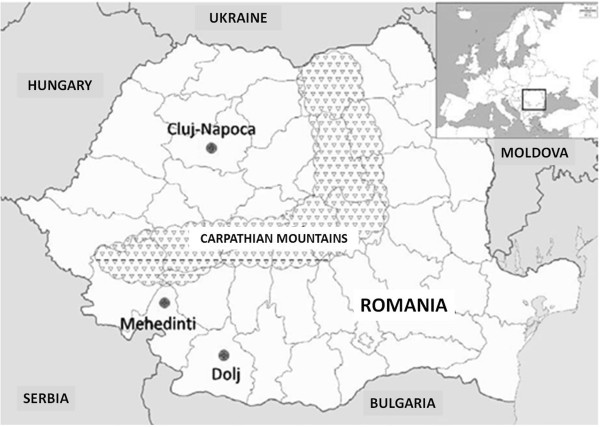
**Geographical location of the two Romania populations analyzed in the present study.** North Romania (Cluj-Napoca) and South Romania (Dolj and Mehedinti).

The present population of Romania is homogenous from a cultural and linguistic point of view, with an absence of language dialects within the borders of the country despite the relatively large and diverse geographic areas inhabited. Several historical events impacted the demography of Romanian populations inside and outside the Carpathian range. In this regard, although relatively little is known about potential differences in the Neolithic and the Bronze age, an impact of Celtic migration has been reported mainly in Transylvania (inside the Carpathian range), rather than in the planes East and South of the mountains [8]. During the Iron age and Antiquity the population was represented both inside and outside the Carpathian range by Geto-Dacian tribes, later incorporated in the Roman Empire after the conquest of Dacia by emperor Traianus in 106 AD. Several waves of migrations from both West (Gothic tribes) and East (Huns, Slavs, Magyars, Cumans) have differently impacted the country. While the Middle Ages were characterized by Hungarian and Germanic influences in the territory West of the Carpathians. The Greek and Turkish influences dominated in South and East of the country [9]. The unity of Romania was realized only during the 19th - 20th century, and was completed after the First World War. Therefore, while Romanians are seen in genetic and medical studies as one homogenous group, potential differences may have important epidemiological consequences. In order to test the hypothesis that the Carpathian mountains represents a genetic barrier differentiating populations within Romania, we have assessed mtDNA distributions in Romanian populations from outside (South Romania) and within (North Romania) the Carpathian range (Figure 1).

## Results

The mitochondrial variability obtained in the population from the North of Romania (i.e., within the Carpathian range) (N = 62) was classified in 51 different sequences or haplotypes, which indicates a high degree of sequence diversity (0.9905 ± 0.0072) (Table 1 and Additional file 1). Seven out of the 51 mitochondrial sequences obtained in this population show up in more than one individual, being the rCRS sequence the most frequent one (Table 2). This mitochondrial haplotype variability was classified into ten mitochondrial haplogroups (H, U, K, T, J, HV, W, M, X and A). Haplogroup H was the most frequent (59.7%) while haplogroups M, X and A were the least frequent ones (1.61% each haplogroup). Besides, haplogroups K and HV, show up also at a low frequency (3.23% each haplogroup) (Table 2).

**Table 1:** Sequence and haplogroup diversity indices for mtDNA lineages in North and South Romania populations

	**North of Romania**	**South of Romania**
N. individuals	62	66
N. sequences (K)	51	43
Sequence diversity (Ĥ ± sd)	0.9905 ± 0.0072	0.9804 ± 0.0059
Nucleotide diversity (π ± sd)	0.01180 ± 0.00089	0.01068 ± 0.00082
N. polymorphic sites (s)	58	51
N. haplogroups	10	7
Haplogroup diversity	0.7542 ± 0.0461	0.6256 ± 0.0622

**Table 2:** Distribution of the frequencies (%) of the mtDNA sequences (HT) and haplogroups (HG) obtained in North Romania population

**HT**	**HT(N)**	**HT (%)**	**HG**	**HG (%)**
rCRS	5	8.06	H	
16070G	3	4.84	H36	
16189C 16356C 16362C	2	3.23	H1b	
16311C	2	3.23	H2b	
16092C 16293G 16311C	2	3.23	H11	
16129A	1	1.61	H	
16093C	1	1.61	H	
16153A	1	1.61	H	
16354 T	1	1.61	H2a1	
16357C	1	1.61	H	
16092C 16189C	1	1.61	H	
16209C 16261 T	1	1.61	H	
16304C 16311C	1	1.61	H5	
16093C 16129A 16207G	1	1.61	H	
16241G 16311C	1	1.61	H	
16093C 16249C 16362C	1	1.61	H6	
16278 T 16293G 16311C		1.61	H11a	
16080G 16183C 16189C 16223 T 16356C	1	1.61	H1b	
16162G	1	1.61	H1a	
16304C 16311C 16391A	1	1.61	H5	
16093C 16223 T	1	1.61	H	
16362C	1	1.61	H6	
16288C 16311C 16362C	1	1.61	H8	
16148 T 16256 T 16319A	1	1.61	H13a2	
16287 T	1	1.61	H12	
16189C 16304C	1	1.61	H5	
16093C 16311C	1	1.61	H	59.68
16075C 16256 T 16270 T 16399G	1	1.61	U5a1	
16189C 16270 T	1	1.61	U5b1	
16093C 16189C 16270 T 16301 T	1	1.61	U5b1b	
16146G 16342C	1	1.61	U8a1	
16169 T 16356C	1	1.61	U4	
16051G 16092C 16129C 16183C 16189C 16362C	1	1.61	U2	
16189C 16192 T 16256 T 16270 T 16291 T 16399G	1	1.61	U5a1	
16256 T 16270 T 16399G	1	1.61	U5a1	11.29
16224C 16311C	2	3.23	K	3.23
16126C 16294 T 16296 T 16304C	2	3.23	T2b	
16126C 16163G 16186T 16189C 16294 T	1	1.61	T1a	
16126C 16294 T 16296 T 16362C 16390A 16399G	1	1.61	T	6.45
16069 T 16126C 16261 T	1	1.61	J1	
16069 T 16126C	1	1.61	J	
16069 T 16126C 16189C	1	1.61	J	4.84
16298C 16362C	1	1.61	HV0	
16298C 16311C	1	1.61	HV0	3.23
16223 T 16292 T 16399G	1	1.61	W	
16223 T 16292 T 16355 T 16362C	1	1.61	W	
16147 T 16223 T 16286 T 16292 T	1	1.61	W	
16172C 16223 T 16231C 16292 T	1	1.61	W	6.45
16129A 16223 T 16291 T 16298C	1	1.61	M5	1.61
16183C 16189C 16223 T 16255A 16278 T	1	1.61	X2c	1.61
16039A 16188 T 16189C 16223 T 16290 T 16319A 16356C 16362C	1	1.61	A	1.61

The mitochondrial variability obtained in the population from the South of Romania (N = 66), was classified in 43 different sequences or haplotypes, which indicates also a high sequence diversity (0.9804 ± 0.0059), but lower than that obtained in the sample from the North of Romania (Table 1 and Additional file 1). Thirteen out of the 43 mitochondrial haplotypes obtained in this population appear in more than one individual (Table 3). As in the sample from the North of Romania, the rCRS haplotype was also the most frequent one. This mitochondrial variability was classified within seven mitochondrial haplogroups (H, U, K, T, J, HV and W). Haplogroup H showed the highest frequency (47%), although it was lower than the frequency of haplogroup H in the sample from the North of Romania (59.7%). Haplogroup U showed a noticeable frequency (17%), higher than in the sample from North Romania (11.3%) (Table 2 and Table 3). As regards haplogroups M, X and A, they were not observed in the South Romanian sample (Table 3).

**Table 3:** Distribution of the frequencies (%) of the mtDNA sequences (HT) and haplogroups (HG) obtained in the population from South Romania

**HT**	**HT (N)**	**HT (%)**	**HG**	**HG (%)**
rCRS	6	9.09	H	
16183C 16189C	5	7.58	H	
16294 T 16304C	3	4.55	H5	
16070G	2	3.03	H36	
16189C 16384A	1	1.52	H5	
16124C 16304C	1	1.52	H	
16209C 16304C	1	1.52	H5	
16182C 16183C 16189C	1	1.52	H	
16188G	1	1.52	H	
16291 T 16343G 16390A	1	1.52	H	
16042A 16288C	1	1.52	H1	
16093C 16189C	1	1.52	H1	
16113C 16311C	1	1.52	H	
16343G 16390A	1	1.52	H	
16189C 16384A	1	1.52	H5	
16139 T	1	1.52	H	
16093C 16311C	1	1.52	H	
16183C 16189C 16356C 16362C	1	1.52	H	
16189C 16311C	1	1.52	H	46.97
16356C	3	4.55	U4	
16256 T 16270 T 16399	2	3.03	U5a1	
16051G 16179T 16356C 16362C	2	3.03	U4c	
16197 T 16256 T 16270 T	2	3.03	U5	
16179 T 16356C	1	1.52	U4c	
16192 T 16256 T 16270 T	1	1.52	U5a	16.67
16224C 16311C	2	3.03	K	
16224C 16311C 16360 T	2	3.03	K2a	
16224C 16311C 16362C	1	1.52	K	7.58
16182C 16183C 16189C 16294 T 16296 T	1	1.52	T2	
16126C 16294 T 16324C	1	1.52	T	
16126C 16163G 16170G 16186 T 16189C 16323 T 16294 T 16316G	1	1.52	T	
16126C 16193C 16294 T 16324C	1	1.52	T	6.06
16069 T 16126C	2	3.03	J	
16066G 16069T 16126C 16145A 16172C 16261 T 16311C	1	1.52	J1b	
16069 16126C 16261 T	1	1.52	J	
16069 T 16126C 16224C	1	1.52	J1c	7.58
16298C	3	4.55	HV0	
16067 T	1	1.52	HV0	
16067 T 16126C	1	1.52	HV0	
16147 T 16221 T	1	1.52	HV4	
16298C 16311C	1	1.52	HV0	10.61
16223 T 16286 T 16292 T 16311C	2	3.03	W	
16051G 16189C 16192 T 16223 T 16292 T 16325G	1	1.52	W	4.55

**Figure 2:**
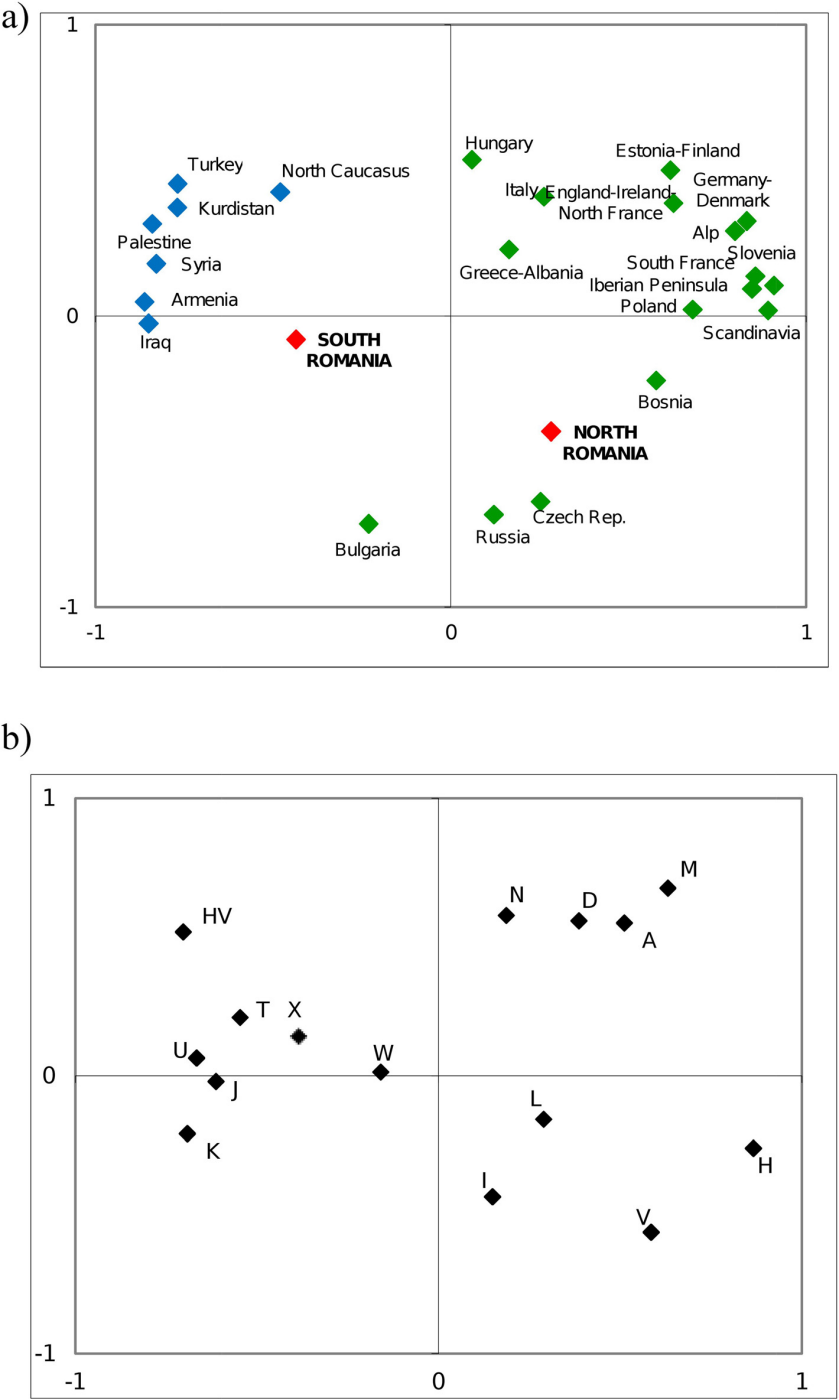
**Principal Component Analysis (PCA) (57% of total variance in two First Principal Components). a)** Distribution of the populations based on the frequencies of the mitochondrial haplogroups of North and South of Romania (in red), Europe (in green) and Near East (in blue). **b)** Correlation of the mtDNA haplogroups with the main axes of the PCA.

After performing the pairwise F_ST_ test (Additional file 2) based on mitochondrial haplogroup frequencies, we did not detect statistically significant differences between the Northern and Southern Romania population samples. When comparing these two samples with other population samples from Europe and the Near East, we observed that Southern Romanians did not show statistically significant differences with any other population. However, the Northern Romanians did show statistically significant differences with the Near Eastern populations (Additional file 2). Regarding the Romanian populations (North and South of Romania) statistically significant differences were detected when the comparison was based on haplotype frequencies (p = 0.00000 ± 0.0000, pairwise F_ST_ test). This could be due to the fact that North and South Romanian samples share only eight mitochondrial haplotypes. On the other hand, the analysis of the mitochondrial haplotype variability obtained in Romanian samples in the context of the Near Eastern and North East European populations, showed that 30 out of 51 haplotypes (58%) obtained in the North Romanian sample have been found only within the populations of North East Europe, and 21 out of 51 haplotypes (41%) have been found in both North East Europe and the Near Eastern populations. Regarding the Southern Romanian sample, 16 out of 43 haplotypes obtained (37%) have been found only in the Near Eastern populations, and 10 out of the 43 haplotypes obtained (23%) have been found in both the Near East and North East European populations.In the First Component (43% of the variance) of the Principal Component Analysis (PCA) (57% of total variance in two First Principal Components) (Figure 2), the European populations lie at one end of this axis, whereas the populations of the Near Eas tare located at the other end of this axis. The population of Northern Romania is within the range of the mitochondrial distribution observed for the other European populations, while the distribution of South Romanians was closer to that of the Near Eastern populations.

The mitochondrial haplogroups that show the higher value of correlation with the First Component are haplogroups H, HV and K (correlation coefficient 0,867, −0,703, −0,691 respectively) (Figure 2). Thus, the different position of North and South Romanian populations in the First Principal Component is explained by the distribution of the frequencies of haplogroups H, HV and K. In this regard, we observe that the North Romanian population is located alongside with other European populations because it has a high frequency for haplogroup H (59.7%) and low frequencies for the haplogroups HV and K (3.23% each haplogroup). On the contrary, the population of South Romania has a lower frequency of haplogroup H (46.9%) and higher frequencies of the haplogroups HV and K (10.61 and 7.58%, respectively); the frequency distribution of these haplogroups in South of Romanian is in the range of variability of some Near East populations [10].

The Bulgarian, Hungarian, Russian and Czech populations are located between the North and South Romanian populations in this First Component. Bulgaria shows more similarity with South Romania than with North Romania regarding the frequency distribution of haplogroups K (7.5%), HV (6%) and H (43%) [11]. However, the samples from the Czech Republic and Russia, show frequencies for haplogroups HV (4.2 and 1.6%, respectively) and K (2 and 3.9%, respectively) [12-14] that are closer to the frequency values described in the North Romanian sample (Figure 2).

The Second Component of the PCA, which explains 14% of the variance, did not show a clear clustering of populations. The haplogroup with a higher correlation with this Second Component is haplogroup M (correlation value 0.677). The populations from Northern Romania, Czech Republic, Russia and Bulgaria differ from the rest in their high frequency for haplogroup M (4.3-0.9%) [11-14], whereas haplogroup M is absent or very rare in other European and Near Eastern populations (Figure 2).

**Figure 3:**
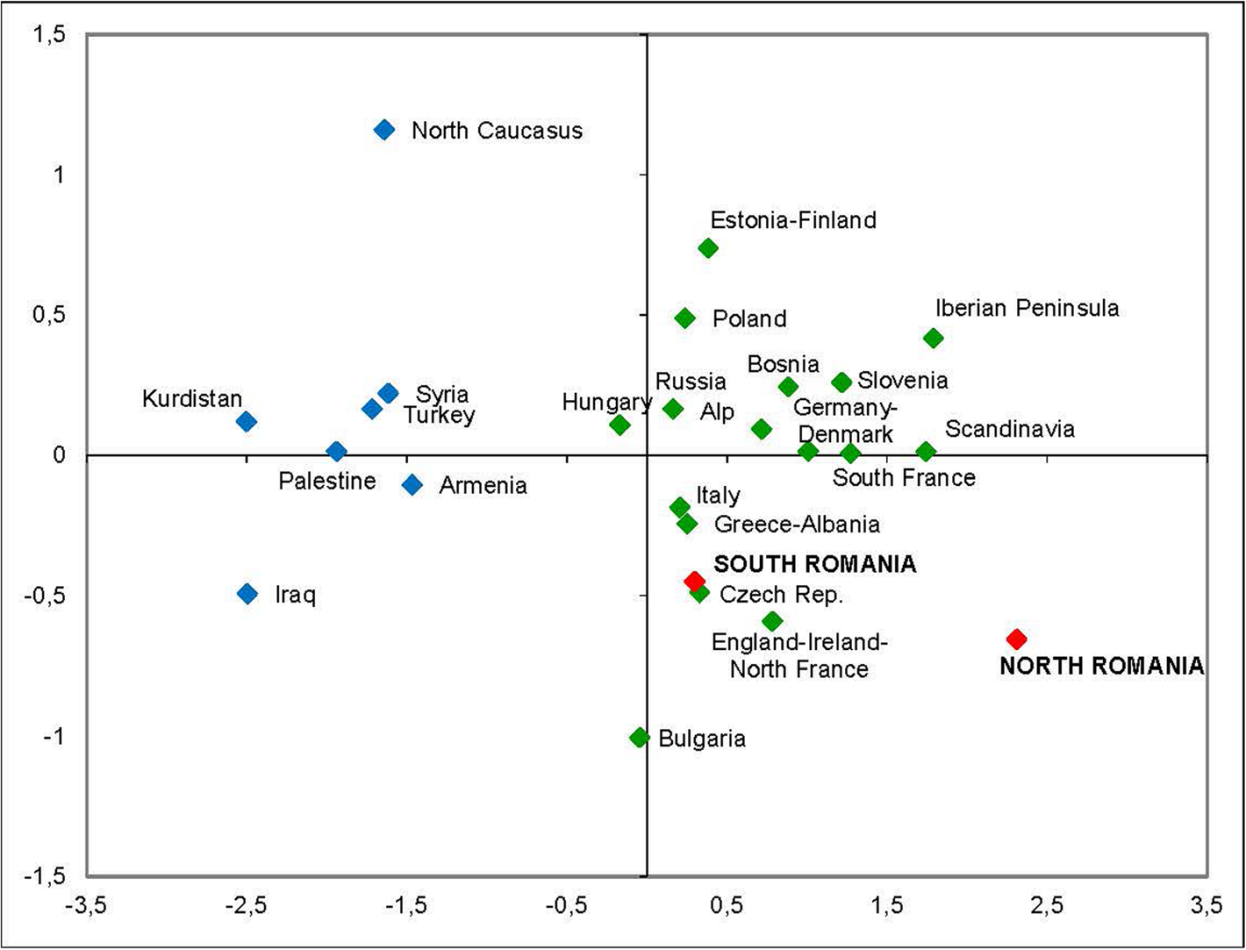
**Multidimensional Scaling (MDS) based on the F**_**ST **_**genetic distances, calculated according to the distribution of the mtDNA haplogroup frequencies of different populations: North and South of Romania (in red), Europe (in green) and Near East (in blue).**

**Figure 4:**
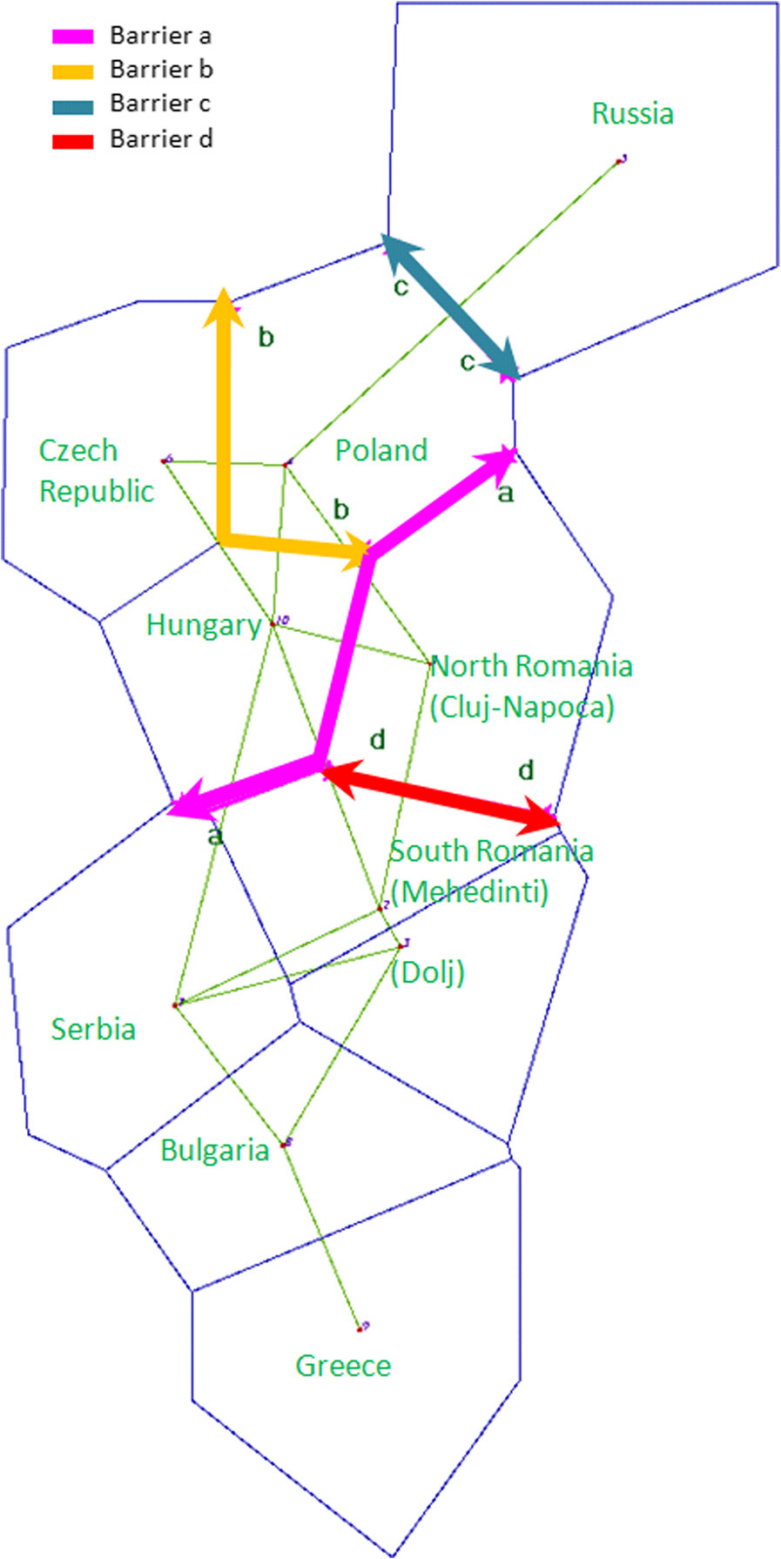
**The first four genetic boundaries (lines: a, b, c and d) detected by BARRIER version 2.2 using genetic distance matrix based on the mtDNA haplogroup frequencies.** The Romanian groups from the North and South (present study) have been considered together with the surrounding populations.

A Multidimensional Scaling (MDS) analysis, where all mitochondrial variability was taken into account, was also carried out to provide a two-dimensional view of the *F*_*ST*_ distance matrix. The analysis showed an RSQ of 0.98037 and a stress value of 0.07343, which indicates that the representation of the MDS obtained showed a good description of the real mitochondrial variability. In the MDS analysis, the Near Eastern and the European populations presented the greatest distance (Figure 3). Moreover, the analysis showed that the South Romanian population was located within the mitochondrial variability of the European populations, whereas North Romania is separated from the South Romanian population.In the MDS (Figure 3), the location of the Eastern European populations including North and South Romania, Bulgaria, Hungary, Czech Republic, Russia, Slovenia and Poland showed a heterogeneous distribution, without a clear clustering. In addition to that, the Czech and Russian populations were located closer to South Romania than to North Romania population, in contrast with the position showed in PCA analysis (Figure 2).The analysis of the genetic boundaries between the populations around Romania supported the four boundaries shown in Figure 4. The first boundary [(a), with 93% bootstrap support] was found between Hungary and both Romania (North and South) and Serbia. The second boundary [(b), with 83-92% bootstrap support] was found between Poland and both Hungary and Czech Republic. The third boundary [(c), with 82% bootstrap support] separated the populations of Russia from Poland. Finally, the fourth boundary [(d) with 80% bootstrap support] was found between North and South Romania.

**Figure 5:**
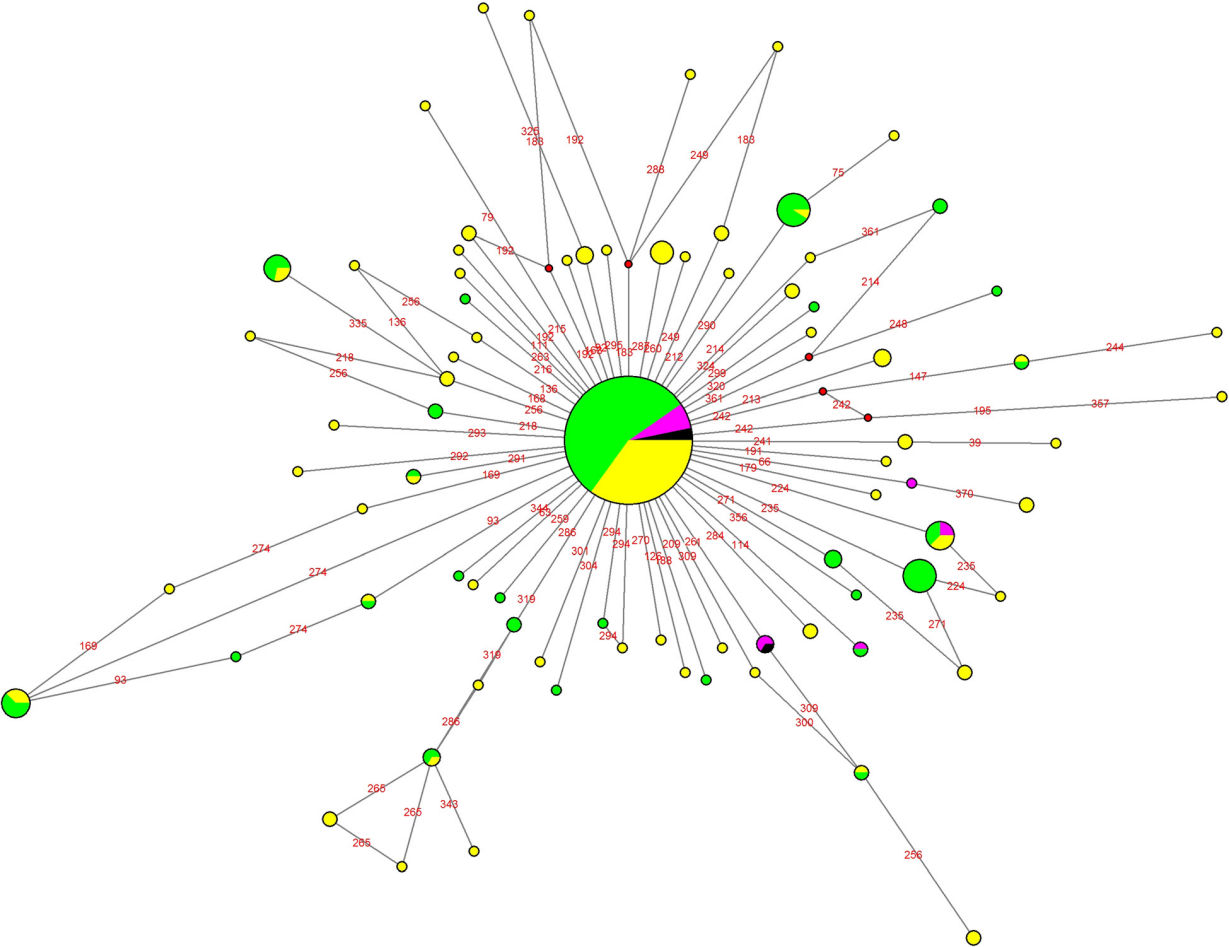
**Median Joining Network of haplogroup J. Data encompass mtDNA HVS-I (nps 15999–16399).** South Romanian sample in pink, North Romanian sample in black, Near East populations in green and North East Europe population in yellow.

**Figure 6:**
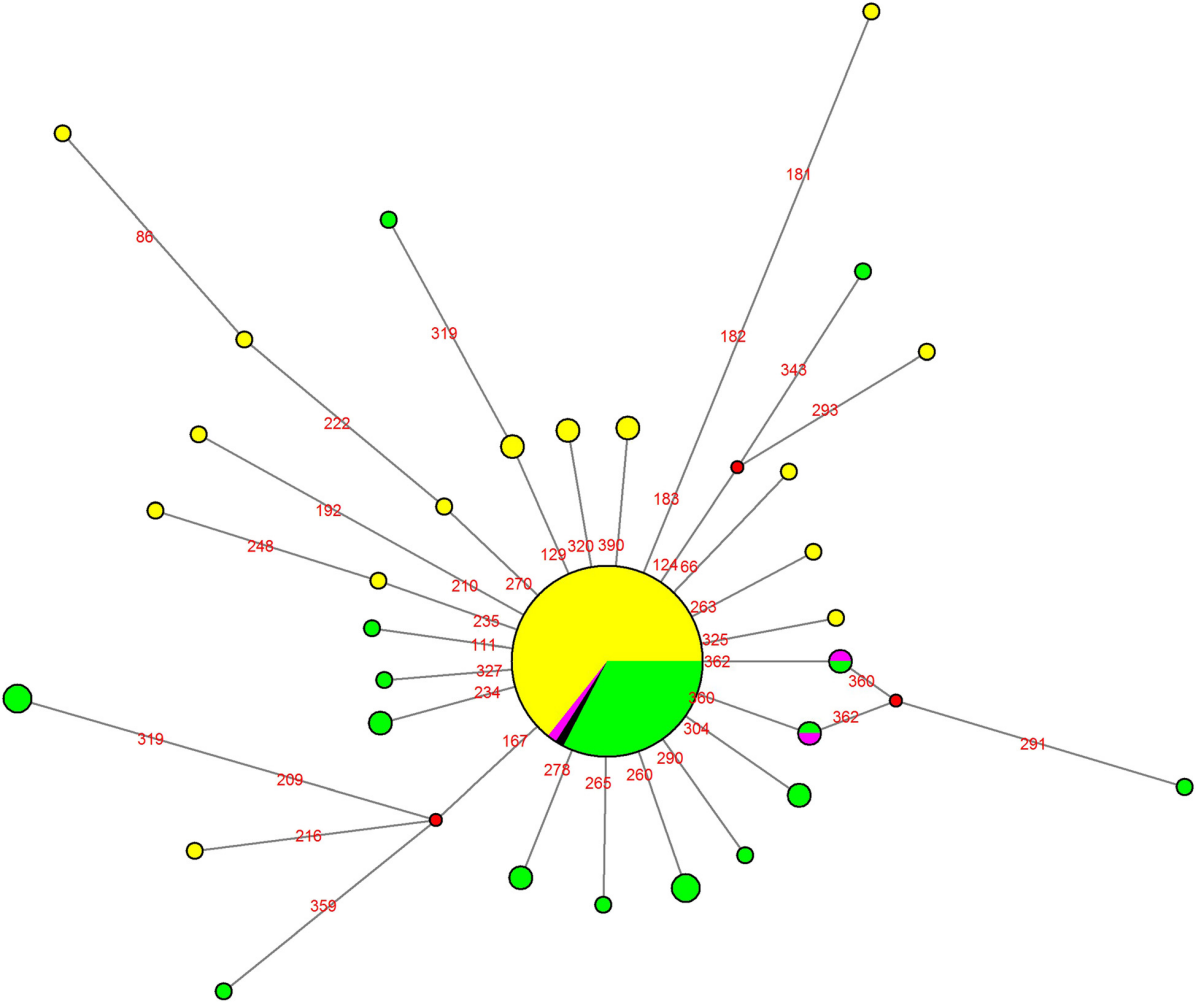
**Median Joining Network of haplogroup K.** Data encompass mtDNA HVS-I (nps 15999–16399). South Romanian sample in pink, North Romanian sample in black, Near East populations in green and North East Europe population in yellow.

Next, we decided to obtain Median Joining Networks (MJN) for haplogroups J (Figure 5) and K (K2a) (Figure 6) because these haplogroups are considered as markers of the Neolithic expansion into Europe from the Near East [10,15,16]. For this MJN analysis, the HVS-I sequences of the North and South Romanian samples obtained in this study, have been considered together with a set of sequences of North East European and Near Eastern populations. The resulting MJN for haplogroup J and K (K2a) are shown in Figures 5 and 6, where the central node is represented by the polymorphisms which define each haplogroup, i.e. 16224–16311 in the case of haplogroup K and 16069–16126 for haplogroup J.In the case of haplogroup J (Figure 5), the MJN central node is defined by polymorphisms 16069–16126, which are shown by all of the samples. On the one hand, it can be observed the high sequence diversity within this haplogroup, and on the other, that the haplotypes of haplogroup J are more common in the Near East population than in North East European populations. With regard to the Romanian samples, it can be highlighted that those sequences found only in the South Romanian sample are also found within the Near Eastern populations.Regarding the MJN of haplogroup K (K2a), haplotype 16224–16311, located in the central node (Figure 6), has been described in both South and North Romanian groups, as well as in the North East European and Near Eastern populations. However, two haplotypes defined by polymorphisms 16224-16311-16360 and 16224-16311-16362 are shared only by one individual from the Near East population and a few individuals from the South Romanian sample.

## Discussion

In the present study we carried out an assessment of the mtDNA variability of Romanian populations from outside (South Romanians) and within (North Romanians) the Carpathian mountain range, in order to assess its influence as a potential genetic barrier on the genetic variability of Romanians.

The North Romanian population exhibited several differences in the frequency distribution of certain mitochondrial haplogroups comparing with other populations. Haplogroup H showed a very high frequency (59.7%) (Table 2), while in Europe the frequency of haplogroup H reaches 42-55%, and in the Near East this is substantially lower, 23-33% [10]. Haplogroup U in North Romania showed a slightly lower frequency (11%) than in most European (13-19%) and Near Eastern populations (21-27%) [10]. Furthermore, in the North of Romania, a low frequency of haplogroup J was found (4.8%) compared with that observed in other populations of Europe and the Near East (Table 2, Figure 5).

The frequency of the remaining mitochondrial haplogroups in the North Romanian sample (K, T and HV) were within the European mitochondrial haplogroup frequency variation, except for haplogroups M, X and A, which are very rare in Europe. Haplogroups X and A are found mostly in Eastern Asia and America, and haplogroup M is the root of many of the haplogroups derived from the dispersion of *Homo sapiens* out of Africa, and it can be found mainly in Southern Asia, having a high frequency in Indian subcontinent [17]. The presence of these haplogroups in North Romanians, albeit at low frequencies, might be the result of the early medieval migrations of Asian populations of Huns, Avars, Magyars and Cumans that crossed the territory of Transylvania between the 5th and 11th century AD.

The mitochondrial haplogroup variability (H, U, J, K, T and W) of the sample from Southern Romania is, in general terms, within the range of variation of other European populations, except for haplogroups HV and K. The frequencies of haplogroup HV (10.6%) and K (7.6%) are closer to the range of variation described in the Near East (7-17% and 5–10.8%, respectively) compared with other European populations (0-7% and 2–6.2%, respectively) [10]. Regarding haplogroup K, the distribution of the haplotypes shown in the MJN is more similar to that of the Near East than to that of the European populations (Figure 6).

Therefore, the frequency distribution of mtDNA haplogroups in Romania indicates certain differences between the North and the South of the country (Table 2 and Table 3). Although the F_ST_ analysis based on haplogroup frequencies did not indicate statistically significant differences between North and South of Romania, the pairwise F_ST_ analysis based on mitochondrial sequence frequencies showed statistically significant differences between the two populations. The Northern Romanian haplogroup distribution is statistically significant different from that observed in the Near Eastern populations, while the haplogroup distribution from Southern Romania does not present such differences (Additional file 2). These results could be due to the fact that North Romanian sequences were closer to the sequences from North East Europe, but in the case of the South Romanian sequences, a better match to the Near East populations was observed.The distinction between North and South Romanian populations is also appreciated in the PCA where the South Romanian population is located closer to the range of variation of the Near Eastern populations, whereas the sample of Northern Romania is located within the range of European variation (Figure 2).

According to the First Component of the PCA (Figure 2), the distinction between the South and North of Romania populations can be explained by a different influence of Near Eastern populations. The South of Romania, as well as the Bulgarian population, presents a high frequency for haplogroup K; moreover subhaplogroup K2a, proposed as a possible marker of the dispersion of farming from the Near East [10,15,16], has been found in both samples (Tables 2 and 3). On the contrary, the North Romanian population (as well as Russia and the Czech Republic) did not show any individual belonging to haplogroup K2a, and the frequency of haplogroup K is in the range of variation of other European populations. Regarding the Second Component of the PCA, the samples of Bulgaria, Russia and Czech Republic can be clustered with the North Romanian sample due to the presence of haplogroup M (4.3-0.9%), which is absent or very rare in Europe and Near East, having higher frequency in Western and Southern Asia.

Therefore, the distribution of mitochondrial haplogroups in North and South Romania and their neighboring populations (PCA, Figure 2) cannot be explained by a North–south differentiation, but most likely due to a differential genetic influence in Europe from Near Eastern populations. The differential influence of population movements from the Near East in Romania can be detected by the presence of higher frequency for the haplogroup J and haplogroup K2a in the sample of southern Romania (Figure 5 and Figure 6). These haplogroups, given their coalescence age, were considered as markers of the Neolithic expansion in Europe from the Near East [10,15,16].Despite these differences between North and South Romania, when a MDS analysis encompassing all mitochondrial variability of the populations (Figure 3) is considered, it appears that both Romanian populations are included within the range of the European mitochondrial variability, rather than being closer to the Near Eastern populations. However, the North Romanian population is slightly separated from the rest of the populations included in the (MDS) (Figure 3). Interestingly, the results of the Monmonier’s algorithm (Figure 4) showed four genetic boundaries in Eastern Europe, with the Carpathians range being the weakest of them.

The mtDNA differences between North Romania and South Romania may reflect the fact that they suffered a different genetic impact of past demographical events from prehistory to the present. Thus, we suggest that the Carpathian mountain range that crosses the country would act as a weak geographical barrier partially limiting the contact between the two Romanian regions. However, this limitation is weak at most, without a strong Northern and Southern Carpathian Mountains mitochondrial haplogroup differentiation. Instead, the haplogroup composition of South Romania reflects the trace of the demographic movements that also affected other Southern European populations. In contrast, the mitochondrial haplogroup diversity found in North Romania may reflect other prehistoric (Celtic influence) or historic (Eastern Asian migrations) events that differentiate the region of Transylvania compared to the rest of Romania.

## Conclusions

In the present study we evaluated whether Carpathian Mountain represent a genetic barrier in East Europe. Regarding the mtDNA haplogroup and haplotype distributions the populations living *outside* the Carpathian range (South of Romania) displayed some degree of genetic differentiation compared to those living *within* the Carpahian range (North of Romania). However, this differentiation can be mostly attributed to the demographic movements from East to West (prehistoric or historic) that differently affected in North and South Romania.

## Methods

### Populations

In the present work, we analyze the mtDNA variability of 128 individuals from two different Romanian regions separated by the Carpathian mountains, including 62 samples from Cluj-Napoca (North of Romania) and 66 from Dolj and Mehedinti (South of Romania) (Figure 1). Selection criteria included the Romanian origin of the donors, and the geographical origin of their parents and grandparents within the Romanian region. Participants were carefully selected by the authors in the field to avoid inclusion of relatives in the sample. The study was approved by the Craiova University Ethical Committee, we have indeed obtained written informed consent from all the volunteers, and none of them were children.

### Mitochondrial DNA analysis

The maternal ancestry of the 128 Romanian individuals was explored by the *D-loop* region sequence variability, including the analysis of the HVS-I (nts 16,000-16,399, as per Andrews [18]. In the individuals with an HVS-I haplotype corresponding to more than one possible haplogroup, we analyzed the sequence of HVS-II (nts 1–425, as per Andrews [18]). Likewise, in order to verify the mtDNA haplogroups obtained, nucleotide positions of the coding region were determined by means of PCR-RFLPs, as described in Izagirre and de la Rua [19].

PCRs were performed in 25 μl of reaction mixture containing 10 mM Tris–HCl pH 8.3, 2 mM of MgCl_2_, 0.1 μM of each dNTP, 0.4 μM of each primer, 5 units of Taq (Bioline) and 10 μl of diluted DNA (1 μl of DNA extract in 10 μl of distilled water). Cycling parameters were 95°C for 5 min, followed by 35 cycles of 95°C for 10 sec, 58°C for 30 sec and 72°C for 30 sec, and a final cicle of 72°C for 10 min. The sequence of the primers to amplify the HVS-I and HVS-II, were those in Hervella *et al.*[20]. In the case of positive amplification and absence of contamination, the amplification products were purified by ExoSAP-IT (USB Corporation), with subsequent sequencing in an ABI310 automatic Sequencer using Big Dye 1.1 chemistry (*Life Technologies*). The results obtained were edited with BioEdit (http://www.mbio.ncsu.edu/BioEdit/bioedit.html) and the sequences were manually aligned.

Finally, in order to classify the mitochondrial variability of the individuals analyzed in this study, we proceeded to amplify 11 markers to define the 10 Western Eurasian haplogroups [21]. The protocol and primers are described in [19,22]. The digestion patterns were analyzed using a fragment Bioanalyzer (Agilent Technologies).

#### Statistical analysis

Intrapopulation genetic diversity parameters such as the number of different sequences (K), sequence diversity (Ĥ) [23], number of polymorphic sites (S) and nucleotide diversity (π) [23,24] were calculated for the HVS-I using the DnaSP software v5.10.01 [25] and the Arlequin software v3.11 [26]. The genetic distances (F_ST_ distances) were calculated on the basis of haplogroup and haplotypes frequencies using Arlequin software v3.11 [26]. In addition, we have analyzed the mtDNA variability of both Romanian samples in the context of other European and Near Eastern populations [10-14,27-50] by means of Principal Component analysis (PCA) (SPSS 17). The distance matrix between all the populations was calculated by means of Arlequin v3.11 [26]. This distance matrix has been depicted in two dimensions by means of a Multidimensional Scaling (MDS) analysis (SPSS 17 Software). Furthermore, a Median Joining Network (MJN) was generated to infer genealogical relationships between the mtDNA lineages (HVS-I) from North and South Romanian, North East Europe and Near Eastern by means of Network software v4.5.0.0 (http://www.fluxus-engineering.com/). Given the high mutation rate of HVS-I, the substitution rates obtained by Meyer *et al.*[51,52] have been applied for assignation of mutational weight between 0–10, corresponding the value of 10 to those positions with substitution rates of 0–1, and the value of 0 to those of rates of 4–5.

The genetic barriers associated with each geographical location including in Figure 1 and population were investigated using Monmonier’s maximum-difference algorithm [53] in BARRIER version 2.2 [54].

## Competing interests

The authors would like to declare no competing financial or personal interests in the preparation of this manuscript.

## Authors’ contributions

Conceived and designed the experiments: CR, MGN, MI, SA, NI. Performed the experiments: MH, SA, NI, CR. Analyzed the data: MH, NI, SA, CR, MGN. Contributed reagents/materials/analysis tools: CR. Wrote the paper: MH, CR, MGN. Revised the manuscript critically for important intellectual content: SA, NI, MI. Approved final version to be published: MH, SA, NI, CR, MGN, MI. All authors read and approved the final manuscript.

## Supplementary Material

Additional file 1 The different mitochondrial sequences (nps 16000–16399) obtained in this study as FASTA file. Click here for file

Additional file 2 **F**_**ST **_**analysis: F**_**ST **_**values (upper the diagonal) and *p*-values with standard deviation (p ± sd) (under the diagonal) based on mitochondrial haplogroup frequencies (P < 0.0002, in grey).** Click here for file
